# The Changing Legal Status of Cats in Australia: From Friend of the Settlers, to Enemy of the Rabbit, and Now a Threat to Biodiversity and Biosecurity Risk

**DOI:** 10.3389/fvets.2018.00342

**Published:** 2019-02-18

**Authors:** Sophie Riley

**Affiliations:** Faculty of Law, University of Technology Sydney, Ultimo, NSW, Australia

**Keywords:** free-roaming cats, TNR, enemy of the rabbit, lethal measures, biosecurity

## Abstract

In NSW, free-roaming cats are regarded as one the biggest threats to biodiversity. Yet, at one stage they were classified as “the enemy of the rabbit” and were protected and released in their thousands. The purpose of this article is to examine the changing status of cats in Australia, demonstrating that regulation frequently depends on a narrow set of values based on the usefulness of cats at a given point in time. By the late twentieth century, the status of free-roaming cats had changed from enemy of the rabbit, to threat to biodiversity and then in the twenty-first century, to a risk to biosecurity. Once the status of cats changed from enemy of the rabbit, management practices followed historically-driven pathways that rely on lethal methods, which do not necessarily prioritize efficacy, animal wellbeing, or changing community outlooks. This is reflected in current practice, which gives scant regard to non-lethal processes, such as Trap-Neuter-Release, and in some cases makes the feeding and release of free-roaming cats, illegal. This article argues that regulatory preferences for employing lethal methods, now occur in a society which increasingly questions the efficacy of these measures, as well as the very need to kill. While TNR is unlikely to provide a complete solution to the problem of free-roaming cats in Australia, given the success of TNR among community groups, accompanied by changing societal perspectives, the time has come for regulators to engage with alternative control methods and include them in their suite of official measures.

## Introduction

It is estimated that Australian families house ~3.3 million pet cats, [*Felis catus* (Linnaeus, 1758)] frequently treasured as family members and otherwise protected by a range of laws and policies that proscribe animal cruelty and impose obligations of care^1^. At the same time, Australia also contains large populations of free-roaming cats, with estimates varying from “between 12 and 19 million” (2) to between “2.1 and 6.3 million^2^.” These cats are categorized as “wild” or “feral,” and according to the Australian Department of the Environment, threaten the survival of some 139 native species, resulting in “severe to catastrophic” impacts on Australian biodiversity^3^. While it is not disputed that free-roaming cats predate on native fauna and can also spread toxoplasmosis, the extent of these impacts remains unsettled^4^. Nevertheless, such threats have been used to justify lethal measures, including poisoning, trapping and shooting, leading to contentious debates between environmentalists and animal welfare advocates^5^. One element of the debate questions whether non-lethal processes, such as trap-neuter-release (TNR) have a place in regulatory regimes^6^.

Although free-roaming cats are now targeted for eradication and control, this was not always the case. In the early days of the colony of New South Wales (NSW) cats were valued as a companion animal, as well as for their ability to catch rats and mice (6). By the end of the nineteenth century, cats had also been acclaimed as “the enemy of the rabbit” and released by the thousands in the hope they would control the spread of rabbits^7^. Yet, barely 100 years later, predation by free-roaming cats was officially listed as a threating process to native biodiversity and some jurisdictions currently regard the presence of free-roaming cats as a biosecurity risk^8^.

The purpose of this article is to examine the changing status of cats in Australia and to evaluate how this links to management practices, particularly those that rely on killing. Historical influences are especially significant, keeping in mind the stark comparison between nineteenth-century regulators who attempted to use free-roaming cats to counterbalance damage caused by rabbits, and the regulatory turn-about in the later part of the twentieth century. At present, in common with other unwanted species, the status of free-roaming cats is underpinned by legal classifications, such as, invasive, pest or feral, which provide the triggers and parameters for regulation (7). These classifications invariably lead to reliance on lethal control, normalizing killing, and shutting down discussion on alternative control methods (8).

In one sense, this turnabout is consistent with regulatory patterns emerging from the later part of the twentieth century, that saw introduced species targeted for eradication and control, by listing their impacts as a threatening process, or otherwise making the species subject to eradication and control^9^. However, unlike other introduced species, the cat was also legally protected and deliberately released, with the expectation that it would control rabbits. The protected status achieved by cats has thus far not been replicated by any other introduced animal now classified as a threat or pest, making the study of cats an important topic.

The discussion adopts a qualitative methodology, analyzing and synthesizing historical and contemporary instruments, to identify and evaluate patterns of behavior. Instruments from the nineteenth and early twentieth century largely comprise legislation, proclamations and newspaper reports, while material from the late twentieth century comprises legislation, policy instruments, codes of conduct, strategies, and plans^10^. This analysis is intended to provide a snapshot of standards and principles relating to regimes and to question assumptions that support those regimes. Two themes predominate: first, that regulation frequently depends on a narrow set of values based on the usefulness of cats at a given point in time; and second, that these values promote killing on the supposition that this is an appropriate and effective response in all situations.

The discussion commences by examining how, during the nineteenth century, cats became elevated from friend of the settler to enemy of the rabbit, a classification which fostered the protection and release of cats across Australia. As identified by Dunlap, these perspectives stemmed from a “natural history” approach, where Anglo-settlers understood, and attempted to modify, their environment through observation of cause and effect, hoping that cats would restore balance to nature by ridding the land of rabbits (9). Cats, however, were not effective in this task, yet remained virtually unmanaged until the end of twentieth century.

By this time, understanding the land had evolved from natural history toward ecology, a movement which incrementally integrated scientific discoveries and advances (9). Although this led to better understanding of relationships and dependencies among species, killing individual species continued to form the backbone of regulation (9–11). Parts two and three evaluate these developments in a socio-legal context, not only exposing limitations on settlers' abilities to remodel the land, but also drawing parallels with current practices. The material from the later part of the twentieth century contains more detailed discussion on management practices. This stems from the fact that the impacts of free-roaming cats had attracted attention and were, therefore, subject to more detailed regulation.

Part four of the article evaluates TNR, both in an official capacity, where TNR is largely dismissed, and at the community level, where TNR has achieved localized success. It is argued that official responses to TNR evince a failure to progress beyond entrenched killing patterns, which have scarcely changed from a nineteenth-century emphasis on destroying pest animals^11^. The official stance also persists in the face of debate on transformed social and cultural values that question the need to kill in every situation. The article concludes that while TNR is unlikely to provide a complete solution to the problem of free-roaming cats in Australia, regulators cannot continue to ignore societal calls for more humane treatment of these animals. At the very least, it behooves regulators to engage with alternative control methods and include them in the suite of official measures.

Before commencing the discussion, it is helpful to clarify key terms, such as, wild/feral/free-roaming, stray, and domesticated that are used in the literature. For the purposes of this article, words, and phrases have the following meanings, which have been adapted from the *Threat Abatement Plan for Predation by Feral Cats*, adopted by the Commonwealth Government of Australia: wild/feral/free-roaming cats are cats who “live and reproduce in the wild… and survive by hunting or scavenging [with] none of their need…[being]satisfied intentionally by humans;” stray cats are those found in urban or rural areas and who “may depend on some resources provided by humans but are not owned;” and, domesticated cats are those who are owned and whose “needs are supplied by their owners^12^.” These categories demonstrate an understanding of the breadth of the human-cat relationship and are useful in contextualizing law and policy. However, as discussed in part three of this article, law and policy does not always reflect these subtleties. In addition, cats may move among these categories, further complicating regulation^13^.

## Friend of the Settler and Enemy of the Rabbit: Observations from Natural History

The fortunes of the cat in Australia were closely connected with the introduction of the rabbit, an event which occurred during the nineteenth century and which coincided with land management practices that fostered the introduction and removal of species with impunity.

### Friend of the Settler and Enemy of the Rabbit

Although debate surrounds the manner and timing of the introduction of cats, records indicate that they arrived in 1788, at the time of European occupation^14^. In the early days of NSW, cats were valued for their skill in controlling rats and mice and also as a companion animal, a role that increased throughout the nineteenth century as Australia adopted the European practice of breeding show cats^15^. Both the aesthetic and practical appeal of cats secured their position, so that by the late nineteenth century, cats had spread throughout 90% of the continent^16^. They had become a feature of colonial life, yet for much of the nineteenth century they were not at the forefront of settlers' lives. Cats, for example, were not considered especially advantageous or overly detrimental. Accordingly, they escaped the type of treatment meted out to free-roaming dogs, dingoes, kangaroos, quolls, and wallabies, who were earmarked for destruction because of their perceived danger to humans and/or threat to primary production^17^. As the nineteenth century drew to a close, however, the status of the cat was about to change, its fortunes being dramatically linked with the fortunes of another introduced animal, the rabbit.

As with the introduction of cats, domesticated rabbits were brought to Australia in 1788 (16). However, it was not until 1859 when Thomas Austin released wild rabbits into the state of Victoria that rabbits established themselves and proliferated (16). Their impact on the Australian economy was devastating, prompting inquiries, a Royal Commission, and legislation that imposed obligations on landholders to poison rabbits and build exclusion fencing^18^. Yet, rabbits continued to thrive. Not every landholder had the financial resources to comply with legal obligations, which in any event often proved futile because the crown was not under similar responsibilities, allowing rabbits to move easily from crown land to private landholdings^19^. Economically-based measures, such as bounties were also ineffective because rabbit trappers ensured that a few rabbits always remained, in order to provide themselves with continuous work (17). As these policies collapsed, the damage attributable to rabbits became so great that farmers started leaving their land^20^.

Authorities were impelled to consider alternative measures and they turned to finding the rabbit's natural enemies, who could reduce rabbit numbers to a “natural level,” restoring nature's equilibrium^21^. Accordingly, legislation from 1883 provided that the governor could declare an animal the natural enemy of the rabbit^22^. Once this occurred, the animal became legally protected against killing, capturing or disposal^23^. Numerous declarations were made, evincing strong belief in the restorative power of domesticated and free-roaming cats, “iguanas” (goannas), and “native cats” (quolls) as enemies of the rabbit^24^.

The strength of belief was reinforced by opinion pieces and letters to the editor, as well as by enthusiastic explanations accompanying reports of declarations^25^. One commentary dating from 1892, unequivocally declared “[e]xperience has proved that no damage is done by the cats which confine their attention solely to the rabbits^26^.” While another dating from 1896 noted that although rabbits were increasing in the Dubbo region, “the natural enemies of the rabbit will prove too much for it^27^.” This atmosphere of optimism encouraged the release of cats from NSW in the east to Western Australia in west, leading to the demand for cats quickly exceeding supply^28^. Events at Warrialpha station in South Australia were typical, where the landholder called for the release of additional cats, despite the fact that the station already contained some 5,000 of these animals^29^. Indeed, the notion of cats as an effective bulwark against rabbits persisted into the twentieth century, with one government stock inspector of 18 years' experience declaring that he knew: “…of no more formidable enemy of the rabbit than the domestic cat in a wild state^30^.” Yet, farmers had already observed that notwithstanding how many rabbits were killed, their numbers quickly recovered^31^. In particular, by the early twentieth century commentators observed that decades of killing and poisoning had failed to reduce numbers in the long-term^32^.

Rabbits had learned to avoid poisoned baits, leaving them to be taken up by useful animals such as horses, cattle and sheep, as well as native kangaroos, emus and brush turkeys^33^. In addition, poison destroyed other animals which were enemies of the rabbit, such as goannas, quolls, and cats. This upset the balance of nature by killing the very animals who would otherwise have kept rabbit populations in check^34^. Moreover, notwithstanding the many positive statements regarding the ability of cats to dispatch rabbits, one account from 1891 stated that cats “fraternize” with rabbits and that rabbits often take “no notice of the cats whatsoever^35^.” The writer concluded that cats probably kill a few young rabbits, but “it is evident that the old ones have no fear of them^36^.” This led to a level of dissatisfaction with the failure of official policies, with landholders conceding that rabbit killing was a chronic problem, which would provide regulators with “a permanent job^37^.”

Discontent with official policies was also exacerbated by the fact that, in similarity with poison, cats were destroying animals other than rabbits^38^. In 1863, the famed ornithologist, John Gould observed that cats were attacking and killing a range of native birds and animals^39^. This was consistent with reports elsewhere that cats had killed introduced game birds such as pheasants and partridges^40^, domesticated chickens^41^, small animals and lizards^42^. Cats were also regarded as being especially destructive to sea bird populations on Lord Howe and Macquarie Islands^43^. Moreover, some settlers underscored their concerns by pointing to the fact that cats had no natural enemies, allowing them to multiply “at a great rate^44^.” The role of dingoes or foxes in suppressing cat numbers was mentioned occasionally, but was not seriously discussed, as these were also considered to be agricultural pests^45^.

By the twentieth century, Le Souef, a prominent biologist and zoologist, had expressed misgivings at official policy. He not only drew attention to the impact of free-roaming cats on sea-birds and native animals^46^, but he also questioned the cat's usefulness as the enemy of the rabbit. He pointedly noted that “[the cats'] influence on the rabbit question remains to be seen and it is to be hoped that in this direction they will be of some use to the country and justify their existence^47^.” Almost two decades before these observations, other commentators had voiced comparable concerns, critiquing accepted wisdom that every pest had a natural enemy and noting that “the natural enemies of the rabbits have themselves become pests^48^.” Yet, the notion of managing nature by using natural enemies was hard to shake off and the official position stood firm. In 1913, W E Abbott, an advisor to the NSW government, used mathematical calculations to demonstrate how growing numbers of cats would, in a short time, eradicate any residual rabbits, allowing “the balance of nature…[to] be restored^49^.”

## The Balance of Nature

The balance of nature was an important concept in settler societies. It was intricately connected with ideals of creating “a new England,” to be achieved by dispossessing Indigenous populations and overhauling the land to make it suitable for game hunting, agriculture and pastoral activities^50^. King describes the concept of balance in nature

as a stable state of nature, steadily maintained by the interactions between natural communities and their environment… [allowing] disturbances to this mutual harmony…[to be] corrected by increased attention from their natural enemies…^51^

The ideal balance could be discerned by “observation and common sense,” which would identify predators and prey, encouraging predators to reduce unwanted animals^52^. Cats attacked rabbits, therefore they were the enemy of the rabbit, signifying that more cats meant fewer rabbits. It was a simplistic view that involved limited observation of species' interactions. It did not, for example, deal with broader connections, such as, the impact *of* free-roaming cats on native biodiversity, or the impact *on* rabbits or free-roaming cats of predators, such as dingoes and foxes.

By the twentieth century commentators were making these connections, but they were also aware of the scant regard paid by regulators, which was invariably limited to improving primary production. In 1935, for example, an anonymous letter to the editor of *The West Australian (Perth)*, drew analogies between game management in England and the rabbit problem in Australia^53^. The writer explained that typical management practices involved destroying foxes, cats, and hawks, which were the enemies of game animals, allowing the latter to proliferate. In the writer's opinion, an analogous situation had occurred in Australia. Foxes and raptors were earmarked for destruction because of conflicts with livestock production, yet this ignored the fact that these animals were also the enemy of the rabbit^54^. Accordingly, by killing predators, landholders had upset the balance of nature, allowing rabbits to proliferate^55^. The author, therefore, favored protecting foxes^56^. This was not a novel idea with another commentator having noted in 1923 that although foxes might cause damage during the lambing season, at other times they are “a powerful enemy of the rabbit^57^.” In a similar way, Christopher Lynch who was a rural inspector, had concluded that the presence of foxes meant low rabbit numbers and mooted the idea of protecting foxes (19). The difficulty, however, was that the fox was also an agricultural pest and the thought of it being protected would have been incomprehensible.

In any event, although discussion in the media identified relationships between rabbits, cats, foxes, and native biodiversity, the connections did not filter through to official regulation. In particular, protection of species and subsequently, biodiversity at large, only started gaining momentum from the mid-twentieth century onwards (20). Yet, even at this time, free roaming cats eluded official scrutiny. They did not pose a threat to primary production, nor were they considered harmful to native fauna, thus they escaped regulatory attention.

## Threat to Biodiversity and Biosecurity Risk

Australian jurisdictions have long regulated nuisance/pest/feral animals^58^. However, up to the later part of the twentieth century this was traditionally undertaken to protect the agricultural and pastoral product sectors^59^. The notion of protecting native biodiversity from introduced species started gaining traction after this time. The *Territory Parks and Wildlife Conservation Act 1977* (NT), for example, authorized the Minister to declare species a “feral animal,” triggering obligations on the part of landholders to eradicate declared animals^60^. Several species were categorized as feral, including rabbits, donkeys, pigs, camels, and cats^61^.

It was not until the 1990s, however, with the advent of international interest in the protection of biodiversity that the impact of introduced species, including free-roaming cats, started receiving broad attention. In 1992 Australia became a signatory to the Convention on Biological Diversity, which amongst other things, requires the contracting parties to “prevent the introduction of, control or eradicate” alien species that threaten biodiversity^62^. In accordance with this obligation the Commonwealth Government passed the *Environment Protection and Biodiversity Conservation Act 1999* (CTH) which provides for the listing of threatening and key threatening processes^63^. Predation by free-roaming cats is currently listed as a key threatening process pursuant to this act^64^. Similarly in NSW, Predation by the Feral Cat *Felis catus* (Linnaeus, 1758) was listed as a threatening process in Schedule 3 of the *Threatened Species Conservation Act 1995* (NSW). This has now been carried over to schedule 4 of the *Biodiversity Conservation Act 2016* (NSW). In response to these listings, the state of NSW adopted a range of regional pest management strategies to deal with multiple pests, including cats^65^. At the Commonwealth level, the Australian government has directly targeted free-roaming cats, adopting three threat abatement plans (TAPs): The Threat Abatement Plan for Predation by Feral Cats, 1999 (1999 TAP); The Threat Abatement Plan for Predation by Feral Cats, 2008 (2008 TAP); and, the latest TAP, the Threat Abatement Plan for Predation by Feral Cats, 2015 (2015 TAP)^66^.

In addition to these TAPs and management plans, cats are also managed by an array of legislative and policy instruments that declare them a pest or feral, triggering further eradication and control provisions. In NSW, for example, the *Game and Feral Animal Control Act 2002* (NSW), allows shooting of non-indigenous game animals, defined to include free-roaming cats, as long as shooters have the appropriate license^67^. In the Australian Capital Territory, the *Pest Plants and Animals Act 2005* (ACT) allows the Minister to declare an animal a “pest animal,” which among other things leads to prohibitions on keeping and supplying the animal^68^. At the time of writing, no animal had formally been declared a pest, although authorities have adopted the *ACT Pest Animal Management Strategy, 2012–2022*, a policy instrument that deals with pest animals, including free-roaming cats (22).

The strategy emphasizes the negative impacts of free-roaming cats and proffers a variety of traditional management options based on trapping, shooting and baiting (22). At the same time, the strategy also qualifies the use of lethal methods by noting that cats may not readily accept poison baits and also points out that trapping and shooting are expensive and labor-intensive^69^. Importantly, the strategy stipulates that more research on free-roaming cats is needed and that, apart from trapping and shooting at ecologically important sites, the cat's “[e]cological role as a predator/competitor needs to be determined if a broad-acre control program is contemplated^70^.” These qualifications hint at underlying problems with broadscale lethal control, which in other jurisdictions continues to be rolled out, notwithstanding a lack of adequate data on species interactions and the place of free-roaming cats in Australia^71^. The latest iteration of laws proscribing free-roaming cats derives from biosecurity regulation that encompasses economic concerns, risks posed by cats to human health, as well as threats to biodiversity^72^.

In Queensland free-roaming cats were a declared pest under the *Stock Route Management Act 2002* (QLD)^73^, but are now are regulated under the *Biosecurity Act 2014* (QLD). Amongst other things, the latter contains seven categories of “restricted matter,” which are set out in schedule 2. The categories relate to noxious fish, pest and invasive animals, insects, and weeds and are supplemented by a series of obligations and offenses, that vary according to the category^74^. Typical obligations prohibit the release or distribution of restricted matter, as well as prohibitions on moving or feeding them^75^. Species may be listed in more than one category resulting in overlapping obligations and prohibitions. Free-roaming cats, for example, are listed in categories 3, 4, and 6, leading to prohibitions on feeding or giving them to another person as well as releasing them into the environment. This legislation only differentiates between two categories of cats, domestic cats, and other cats. It does not acknowledge stray cats as a separate category, which has implications, discussed in section Facilitating TNR, with respect to the legality of TNR.

In a comparable manner, Schedule 3 of the *Biosecurity Act 2015* (NSW), lists a number of “prohibited dealings,” which include moving, releasing, feeding or treating domestic cats who have genetic material from *Leptailurus serval*^76^. This prohibition is consistent with a ban on importing savannah cats (a cross between a wild serval cat and a domestic cat) made by the then Minister for the environment, Peter Garrett in 2008 (23). Given the controversy surrounding management of free-roaming cats and the potential for savannah cats to form free-roaming populations, the decision was sound from an environmental perspective, but it proved contentious, as evinced by threats from proposed importers to sue the government^77^.

The restrictions, prohibitions, and control of free-roaming cats discussed thus far, represent views of nature and humanity's relationship to nature, which are based on human ideas of what needs to be protected and how to protect it. Accordingly, in the early days of NSW, cats were defended for their role in destroying rabbits; yet, without a backward glance, the same animal is now targeted for eradication and control. However, some sections of the community are voicing concern about lethal control, raising difficult issues concerning the management of unwanted and problem species^78^. Although advances in science and the scientific method have progressed from natural history to ecology, scientific developments neither dictate how advances in knowledge should be used, nor do they necessarily identify the most appropriate choice of measures^79^. The discussion now leads to the remaining question, concerning community views on lethal control, the effectiveness of TNR and the role of TNR in regulatory regimes.

## Trap Neuter Release

TNR involves catching free-roaming cats, “sterilizing them and returning them to the place where they were found^80^.” It offers regulators a management choice that differs from current practices which rely on wholesale killing in all circumstances. TNR, itself, has led to a lively debate in the literature concerning its practicability, effectiveness and welfare outcomes^81^. In some cases, TNR has reduced population numbers and has had good welfare outcomes, yet in other cases TNR has operated less effectively (25). Australian commentators are ambivalent about TNR, concluding that it “is unsuccessful in open populations and not practical over large areas^82^,” or that it “could work in specific well-defined areas but in general is not a solution to the problem in Australia^83^.” These conclusions, do not give whole-hearted support for TNR; yet they also do not dismiss it out of hand, something that Australian regulation comes close to doing. The background statement to the 2015 TAP states

Capturing, sterilising and releasing (otherwise known as trap, neuter, release/return, or TNR) programs are seen as an effective approach to managing colonies of stray cats in urban areas elsewhere in the world and are promoted in Australia. This approach should be considered unacceptable in Australia as there are no benefits to wildlife and it does not improve the welfare of the individual animals concerned (26)

The 2015 TAP itself, similarly rejects TNR, although it grudgingly concedes that it could be useful in very limited circumstances

The concept of trapping, neutering, and releasing stray cats as a method of population control should also be discouraged on animal welfare grounds and because it is not effective, except where populations are truly isolated and all females are neutered^84^

Notwithstanding the lukewarm appraisal of TNR, two arguments can be made in favor of supporting it, one deriving from management goals and the other based on ethical considerations.

### TNR and Management Goals

Management goals should be a means of aligning activities with aims and objectives. The overarching goal of the 2015 TAP is to minimize the impact of free-roaming cats on native biodiversity^85^. Hence, control and eradication measures should demonstrate improvements in biodiversity protection. In addition, the *Threatened Species Strategy* (TSS) operates in conjunction with the 2015 TAP by detailing policies for species' recovery (27) Both the TSS and the 2015 TAP proceed on the assumption that killing free-roaming cats is the most effective management option^86^. The TSS, in particular, aims at killing 2 million free-roaming cats by 2020, as well assisting in the recovery of 40 threatened mammal and bird species^87^. Yet, neither instrument explains how killing this number of free-roaming cats will improve biodiversity outcomes or aid in species' recovery^88^. By way of contrast, some activities proposed by the TSS, such as reporting and monitoring in the Kosciusko National Park NSW, facilitate gathering and analyzing data, which will identify the effectiveness, or otherwise, of culling^89^. However, the overall focus of the TSS still deems killing *per se* as an effective performance indicator^90^. This outlook is reinforced by a progress report that classifies killing one million cats within 2 years as an important environmental milestone^91^. The literature, however, challenges this comfortable reliance on wholesale killing.

To start with, culling does not always succeed in reducing population numbers in the long-term, unless the number of cats killed “exceed[s] the replacement rate through breeding and immigration” (28). Accordingly, reductions in cat numbers following culling operations are often short-lived, as cats move from adjoining areas to depleted colonies (28). In addition, population numbers are at best, “guesstimates” and where regulators incorrectly gauge the required level of culling it can lead to increased populations^92^. Generally speaking, culling is also unlikely to eradicate free-roaming cats on mainland Australia, a point conceded by the 1999 TAP and confirmed by the 2015 TAP^93^. Managing free-roaming cats is thus likely to remain a lingering environmental problem, creating many regulatory challenges, which to date have not been resolved by continual killing.

Second, the problem also extends to the choice of methods, such as the use of poisons, which kill indiscriminately. “Predation events” are attributable to male cats weighing 3.5 kg or more, signifying that to improve environmental outcomes, poisoning needs to target these animals (30). Yet, free-roaming cats frequently avoid taking poison baits and even when they do, there is no guarantee that individuals responsible for predation will be the ones to do so^94^. In addition, poison destroys other, susceptible animals, including native species that baiting programs are presumed to protect^95^. Regulators are in the process of creating cat-specific poisons, but this also raises ethical issues that are dealt with in the next part of this article.

Third, alternative methods, such as trapping and shooting are more targeted, but they are expensive and not suitable for large areas, although they could be feasible for more restricted areas, such as islands^96^. Yet, even here, the 2015 TAP notes that “[t]his is generally not cost-effective in the long-term” as it still requires continued monitoring and “a sustained control program^97^.” It is therefore a matter of some irony, that cost and ineffectiveness are frequently cited as reasons for dismissing TNR^98^.

Fourth, a related issue stems from species' interactions and the impact of cat eradication programs on populations of rabbits and rodents, which are prey species for free-roaming cats^99^. Research indicates that reducing densities of free-roaming cats would likely lead to increased numbers of rabbits and rodents, which would be an unwelcome side-effect of cat eradication programs^100^. Similarly, the role of free-roaming cats as prey for foxes and wild dogs requires greater consideration^101^. Where populations of free-roaming cats are reduced, the impact on other predators is unclear, particularly whether these predators will turn to native animals. As already discussed, these types of issues were highlighted in the *ACT Pest Animal Management Strategy*, 2012–2022 and are also acknowledged by the 2015 TAP^102^. The latter concedes that while regulators need to be aware of species' interactions, it is a very difficult task, bearing in mind the vastness and variety of ecosystems across Australia and the inconsistency of interactions within these ecosystems^103^. One benefit of TNR is that it does not immediately remove large numbers of free-roaming cats from an environment. Instead, it provides an opportunity to monitor and evaluate changes in the ecosystem as neutered cats die out. From a practical perspective, this fact alone should have signaled that TNR deserves, at least, to be tested properly.

In reality, the reliance on lethal methods is an almost a perverse turnabout of logic, reverting to the natural history approaches of the nineteenth and early twentieth centuries. As discussed, these approaches promoted the removal of unwanted species *per se*, in an attempt to restore balance to nature and also provided the justification for releasing cats to control rabbits. The dissent created by those policies finds parallels with modern-day controversies where environmentalists see culling as the most effective management option, while animal welfarists argue against this. Certainly, arguments made today against wholesale culling of cats, are strikingly similar to those made in the nineteenth and early twentieth centuries against the use of cats to control rabbits: killing does not pay sufficient attention to relationships among species^104^; the ineffectiveness of poison^105^; and, the fact that populations of animals recover as migrations occur from adjoining areas^106^. In as much as the fundamental arguments have not changed, but apply to different aspects of society's relationship with cats, this should cause regulators to question why policy failures of the past are repeated in present-day regimes and why non-lethal methods and ethical concerns have been side-lined.

### TNR, Societal, and Ethical Values

Incorporating ethical concerns, as well as social and cultural values, is essential to managing free-roaming cats. Killing animals polarizes public opinion and without community engagement, regimes may be seen to lack legitimacy. For some, “the only good cat is a dead one^107^.” Yet for others, cats have assumed a high degree of symbolism, being unfairly targeted as scapegoats for loss of biodiversity^108^. Yet again, others with a more pragmatic outlook, agree that regulators need to protect native birds and animals from cat predation, but without relying on wholesale culling^109^. Consequently, cats have social and cultural values that, arguably, should be captured by regimes^110^.

Yet, current policy statements tend to gloss over the importance of societal and ethical values, instead relying on utilitarian ideals to justify culling and the use of poison on the basis that these methods are “net-humane”:

When considering the use of [poison]…, it's important to think about whether it will be effective, and whether the action is justified, including the impact of not taking those actions on the nightly slaughter and maiming of threatened species caused by feral cats. Acting on feral cats is net -humane because it saves millions of native animal lives…It is not realistic or feasible to trap neuter and release millions of feral cats across the more than seven million square kilometres of the Australian continent…[it] would not be humane, effective, or justifiable…. highly stressful for millions of feral cats, transported as wild animals in cages in remote and hot conditions across thousands of kilometres to be neutered and then returned to the wild^111^.

These statements proffer a typical utilitarian analysis that accepts culling as necessary because it is seen as the only way to improve biodiversity outcomes, as well as save native species and deal with the ethical limitations of TNR. The clear message is that TNR raises welfare and conservation issues, which somewhat paradoxically, can only be addressed by dismissing TNR in every situation. In an analogous context, dealing with gray squirrels in the United Kingdom, Crowley et al. conclude that these perspectives make introduced species “killable”

The message is that grey squirrels are not appropriate subjects of care or concern (indeed, some implied that encounters with them shouldn't be encouraged or enjoyed), that their appropriate classification is as vermin or invasives, and that they should be treated (killed) accordingly (32).

The substance of this argument, is consistent with processes that occur in Australia, where national codes of conduct and local management plans turn to killing as a first point response, normalizing it and entrenching it into regulation^112^. Such conclusions are based on underlying assumptions regarding the damage attributable to free-roaming cats, the effectiveness and relative humaneness of culling and the futility of TNR—assumptions which are contested^113^.

To start with, it is important to acknowledge that free-roaming cats can cause environmental harm. A recent study on the damage attributable to free-roaming cats concludes that they impact on native species “through predation, disease transmission, and resource competition…[as well as being] the principal cause of extinction of at least one Australian bird subspecies (Macquarie Island red-fronted parakeet)^114^.” Free-roaming cats have also been implicated in the transmission of diseases such as toxoplasmosis, although the effects on native species are not well-understood^115^. However, free-roaming cats also help control some introduced species, such as rodents and rabbits^116^ and, in addition the scope and extent of threats presented by free-roaming cats remains unsettled^117^. These differing considerations create many challenges for regulators, who must navigate incomplete knowledge structures and community expectations, when deciding on appropriate measures to protect native biodiversity. The latter is without question an important environmental objective, yet the contentious nature of killing makes alternative methods a more palatable solution in the eyes of the public^118^. The intricacies of this point become clearer on further examination of the relationship between killing and the impact of free-roaming cats on biodiversity.

Such impacts vary according to location. In urban and peri-urban areas, for example, cats kill birds, but so too do other predators, such as snakes, goannas, and raptors^119^. This does not necessarily lead to loss of biodiversity, unless more birds are taken than survive to adulthood^120^. Additionally, species taken by cats are invariably the ones who survive urbanization and are often among the most abundant due to increased availability of food and habitat provided by human-generated changes^121^. Perhaps of more concern in urban areas is the way urbanization and land clearing are altering the mix of species, leading to decline in populations of small birds (34–37).

Elsewhere, research reveals that cats usually prey on animals such as rabbits and house mice, while in plague seasons, mice comprise the entire diet of free-roaming cats with rabbits comprising up to “89% by weight^122^.” However, where there are insufficient mammals, free-roaming cats turn their attention to small animals, reptiles, and birds, so that threatened species such as the bilby and marsupial mole may be at risk^123^.

However, it is questionable whether the fact that free-roaming cats threaten native species in some circumstances, justifies the use of lethal measures as the default position on the basis that it is “net humane.” Lethal measures should always require a high degree of justification, and at the very least should be underpinned by sound research that allows them to be deployed where they will be most effective^124^. Moreover, lethal measures need to be monitored, not only to establish whether populations of free-roaming cats have reduced in the long-term, but also to demonstrate how this leads to improved biodiversity outcomes. The 2015 TAP does in fact incorporate provisions regarding research on species interactions and devising ways to improve survival rates of threatened species^125^. However, this needs to be read in conjunction with the TSS that, as already discussed, focusses on killing 2 million cats without providing detail as to how regulators will determine whether culling is linked to successful biodiversity protection^126^.

These matters signal that law and policy rely on a superficial form of utilitarianism that balances killing cats against the assumed ineffectiveness of TNR, as well as unverified biodiversity outcomes. Law and policy do not consider either the pain and suffering of animals who are subjected to lethal measures, or the social and cultural dimensions of free-roaming cats. Although the three TAPs make brief references to ethical, social and cultural concerns, it is doubtful whether these matters are adequately addressed. The 1999 TAP, for example, agreed that regard to “differing cultural values attached to domestic and feral cats [was important to] any control program^127^.” However, this did not lead to cultural values being incorporated into management plans. In a similar way, the 1999 TAP also refers to animal welfare, a concept which has clear ethical implications. Yet, this was seen in terms of a form of “humaneness,” which condoned lethal methods, as long as they were environmentally safe and did not affect domesticated cats^128^. In restricting the notion of humaneness in this way, the TAP deftly side-stepped problematic welfare concerns. The 2008 TAP also contained references to humaneness, but this was equated with the need to develop a toxin-bait that was specific to cats^129^. Likewise, the 2015 TAP acknowledges that ethical and social issues need to be examined, but considers that these issues can be addressed by adhering to the Model Code of Practice for the Humane Control of Feral Cats^130^. However, this code, in common with other model codes, has been critiqued for its focus on lethal measures and lack of ethical awareness^131^.

Consequently, while the TAPs refer to animal welfare, humane methods of control and cultural issues, engagement with these matters is not meaningful. Non-lethal methods, such as using Maremma dogs to protect native species, developing immunocontraceptive vaccines and habitat management, are not given credence^132^. The focus firmly remains on finding a poison that is quick-working, that cats will accept and that is unattractive to non-target animals. This line of thought is so entrenched, that it has extended to investigating whether gene editing can alter cat DNA, to make cats susceptible to particular poisons^133^. It seems incongruous that such a process is being considered, without even a perfunctory review of its ethical basis. From a more pragmatic perspective, these developments also continue to focus on killing, which as already discussed, is not a long-term solution^134^. At best, it is a stop-gap measure that necessitates constant eradication and control efforts^135^. The benefit of TNR is that provides an alternative method that can achieve results and can also re-set the debate by addressing ethical concerns that current regulation side-steps.

Although society might not be conversant with, or even interested in ethical theory, community abhorrence, at mistreating animals has a very practical consequence that manifests in reluctance to endorse killing as the usual response. This much was clear as early as 1913, when in the course of critiquing the effectiveness of cats as the enemy of the rabbit, Le Souef noted that people simply do not enjoy killing cats^136^. In his view, this partially explained why cats continued to be released, rather than being controlled or eradicated, given their ineffectiveness in controlling rabbits^137^. Indeed, disregard of ethical and social values can undermine the best-planned regimes, a point demonstrated by the recent reversal of a planned brumby cull in NSW (39).

In an analogous manner, individuals as well as community groups and animal welfare organizations, in Australian and overseas jurisdictions have undertaken several TNR programs^138^. They have achieved success and have generated important information^139^. At the same time, the programmes in Australia have usually been conducted independently of official strategies, which has meant that the community has had to tread a fine line, to avoid potential breaches of the law as they treat, feed and/or release free-roaming cats.

### Facilitating TNR

The use of TNR among the community is gaining ground. In this context, the notion of “community” is not a term with a settled meaning. It includes programs run by: Non-Government Organizations, such as the Australian Pet Welfare Foundation, which is lobbying for legalized TNR^140^; actions by *ad hoc* rescue groups, such as University of NSW, the Campus Cat Coalition^141^; and, individuals who feed cats, neuter them and return them to the wild (42). In Australia, TNR is commonly carried out by individuals, rather than organizations and occurs in urban areas, such as capital cities (42).

The acceptance and popularity of TNR among the community represents an opportunity for regulators to engage with the public in addressing the issue of free-roaming cats. Indeed, community engagement, is itself an objective of the 2015 TAP^142^. Yet the TAP has interpreted this aim as a call to convince the public that TNR is not viable, warning them against assisting or feeding free-roaming cats^143^. The TAP also advocates managing refuse responsibly, to discourage rats and mice, which are prey for cats^144^. While managing refuse has health and safety benefits, the admonishment against feeding cats raises the prospect of illegality in implementing TNR, both in feeding stray cats and also in treating and releasing free-roaming cats.

The *Prevention of Cruelty to Animals Act 1979* (NSW), for example, simply says that “a person shall not abandon an animal^145^.” This provision, which applies to all animals, potentially makes the release of cats, as part of TNR, an offense under animal cruelty regulation^146^. In Victoria, similar legislation applies to domestic animals or animals “usually kept in a state of confinement for a domestic purpose^147^.” Given that cats can shift between categories, from domestic to stray to free-roaming and back again, these types of provisions create legal uncertainty. Moreover, as already discussed, particularly with respect to Queensland, biosecurity legislation creates offenses for capturing, treating and feeding cats^148^.

Although regulation pertaining to cats may be difficult to police, is susceptible to ambiguities and, as far as the writer is aware, has not yet resulted in any prosecutions, it is nevertheless a formidable barrier to trialing TNR. The threat of illegality and the potential for lawsuits have been identified in other situations as having “chilling” effects, causing stakeholders to waiver in undertaking activities (43, 44). This potentially discourages trials of TNR, a constraint that in a research context is reinforced by the fact that Animal Ethics Committees are highly unlikely to approve research that does not comply with the law^149^. In Australian higher educational institutions, for example, Animal Ethics Committees, are governed by stringent research and integrity policies that specifically call for compliance with rules and regulations^150^.

A novel attempt at dealing with these matters occurred in 2014, when Alex Greenwich, the independent member for the Sydney electorate, introduced a private members bill into the Legislative Assembly of the NSW Parliament. The bill, titled the Animal Welfare (Population Control Programs) Bill 2014 (the Bill), aimed at removing liability for groups and individuals undertaking TNR^151^. In accordance with the Bill, TNR activities would have been licensed and provided they were undertaken under the auspices of a “sponsoring agency,” the activities would not have been illegal^152^. Sponsoring agencies were nominated in clause 3 of the Bill, to include animal welfare organizations such as the Royal Society for the Prevention of Cruelty to Animals, NSW, the Animal Welfare League NSW as well as local government councils. Although the Bill lapsed in February 2015 it nevertheless is instructive.

In a narrow sense, the tabling of the Bill demonstrates that it is possible to draft TNR legislation that conforms with both biosecurity and animal welfare law. In a more general sense, the Bill reflects community concern at the way free-roaming cats are managed. The Bill placed TNR on the official agenda, leading to a parliamentary report on the efficacy of TNR^153^. Although the report dismissed the general practicability of TNR, it did note that in some cases TNR reduced population numbers^154^. The report however, also emphasized that more research was needed to resolve unclear issues, including: whether TNR was suitable only for urban areas; to determine sterilization rates; and, to decide whether community groups should be funded to undertake TNR, with or without, adoption and re-homing programs^155^. These matters, however, are difficult to undertake in the face of uncertainties regarding the legality of TNR. The lapsing of the Bill is also unlikely to stop individuals and community groups proceeding with TNR, although, they need to be creative.

At the University of NSW, the Campus Cat Coalition, manages a colony of cats and kittens who live on property owned by the university. The program, which has run since 2009, is based on spaying/neutering cats, feeding them, vaccinating them, rehoming them where possible, or otherwise releasing them on campus. The Coalition has overcome legal restrictions by claiming ownership of the cats. Research on the project demonstrates that the program has reduced cat numbers, but this is deemed a qualified success, because cat numbers have declined “through adoption of socialized cats and kittens, natural death, or euthanasia of sick animals, and disappearance or emigration of cats^156^.” At the same time, free-roaming cats can emigrate from surrounding areas to the colony, meaning that the effectiveness of the program calls for consistent management and intervention^157^. Notwithstanding these qualifications, the program has succeeded in demonstrating that TNR deserves a role in cat management programs which needs to be further evaluated for effectiveness and community acceptance.

This is not to say that TNR is without challenge. The difficulties just discussed, with respect to cats migrating to managed colonies has also been observed in studies conducted in the United States of America. One survey revealed that population reductions in managed colonies are offset over time by “illegal dumping” of cats and migrations to the colony (46). Other limitations include mixed success rates and the fact that TNR is not suitable for large areas, although it would be feasible for more restricted locations^158^. In addition, TNR generates welfare issues, including the ability of neutered cats to survive in the wild^159^. For this reason, some TNR schemes provide for feeding of cat colonies and also for the removal of individual cats for adoption, or to allow them to be raised in cat sanctuaries (47). Other, practical limitations, stem from challenges in financial and personnel resourcing to feed, house and neuter cats^160^. Nevertheless, TNR still proffers a range of advantages which warrant further discussion.

TNR is arguably more compassionate than lethal methods, such as those that use 1,080, which despite official claims of being net-humane, involves the use of a poison with questionable welfare credentials^161^. Another vital consideration derives from the fact that community TNR projects generate a great deal of information^162^. This material is potentially useful for evaluating the effectiveness of different types of TNR projects and comparing them to regimes based on culling^163^. However, given official antipathy toward TNR, this data can be difficult to collect and verify scientifically^164^. This not only leads to gaps in the information base, but also misses an opportunity to analyse and understand why TNR is effective in some situations and not others. Recent Australian research, for example, concludes that TNR can have positive impacts on population reductions in areas where cats are “over-represented by cat intake into shelters and municipal pounds, and by cat-related complaints^165^.” This observation provides a starting point as to where TNR could be initially trialed. Similarly, studies in the United States of America, have demonstrated that notwithstanding emigration, in the long-term, TNR reduces the size of some cat colonies^166^. Again, this conclusion provides yet another issue suitable for more detailed research. Inasmuch as the literature has identified successful TNR projects, the time has come for large-scale trials of TNR, supported by government funding and regulation that facilitates licensing or exemptions to the law. This would enable the collection and analysis of data to determine whether, and in what way, TNR can be most effective^167^. Accordingly, rather than trying to thwart TNR, government should be resolving legal uncertainties, to facilitate evaluation and consideration of community views conducive to including TNR in its suite of existing measures.

Another, especially important point derives from the fact that as killing wildlife for conservation becomes increasingly common, it also becomes increasingly prone to public scrutiny^168^. As this occurs, the public demands high thresholds of justification for lethal measures^169^. Regimes which ignore or subvert TNR, thus risk alienating the public and undermining the legitimacy of regimes. Increasing scrutiny is particularly pronounced in settler jurisdictions where landscapes have been perceptibly altered by the introduction of species, which are now targeted for eradication and control^170^. On one level, this may be seen as an environmental issue, where lethal measures are necessarily undertaken to protect native biodiversity. Yet, on another level it presents as an ethical dilemma pertinent to how humans ascribe value to animals (49). In this instance, discourse from the field of critical animal studies questions the fact that introduced animals bear the brunt of environmental management, while simultaneously ignoring the significant ethical dimension that those very regimes engender^171^. These points signal that regulators need to find more ethical and workable alternatives to wholesale killing.

## Conclusion

This article was not intended to afford definitive solutions to how to deal with free-roaming cats, but to question assumptions upon which the current regime is based and to argue in favor of creating regulatory space for TNR.

The current focus on killing free-roaming cats evolved from two events in Australia's history: the damage to pastoralism caused by rabbits and the biodiversity crisis of the later part of the twentieth century. In just over 100 years, from the end of the nineteenth century to the beginning of the twenty-first century, cats in Australia have been categorized and re-classified from friend of the settler, to enemy of the rabbit and finally, a threat to native biodiversity and biosecurity. These changes primarily derive from society's relationship to its environment, finding expression in law and policy that has either advanced or opposed the presence of cats in tandem with their perceived usefulness or threat. Up to approximately the middle of the twentieth century, such decisions were made within a natural history framework, identified by Dunlap as a way of understanding the land through direct observation. It was an approach based on simplistic views of cause and effect, which validated the introduction and removal of animals at will. Accordingly, cats were released by the thousands, in the hope that they would keep rabbit populations under control and restore balance to nature. Cats proliferated and although they did not live up to their human-imposed expectations, they remained unregulated for decades.

In the interim, scientific discoveries and advances in ecology provided regulators with detailed understanding of species interactions. As a result, the concept of balance in nature fell into disfavor as regulation aimed for holistic environmental protection, involving habitats, ecosystems, and biodiversity at large. Notwithstanding such advances, free-roaming cats are managed in the shadow of natural history approaches. Cats are earmarked for eradication and control, without adequate regard for species' interactions, or consideration whether culling will lead to improved environmental outcomes. The Australian example is instructive, where strategies and plans aim at culling two million free-roaming cats by 2020, yet lack detail as to how this will improve biodiversity objectives. As with the days of natural history, killing is the mainstay, an approach which has persisted, notwithstanding its long-term ineffectiveness and notwithstanding society's increasing unease at regimes that lack an ethical mainstay.

Although official regulation warns against it, TNR has been gaining traction among the community. However, unlike culling, which is officially sanctioned, the legal status of TNR is precarious. It is discouraged both by government policy and legislation. Indeed, the latter creates offenses for feeding, treating and releasing cats, activities traditionally associated with TNR^172^. Yet, the community continues to find ways to implement TNR projects.

From a regulatory perspective, the official aversion to TNR means that regulators are missing opportunities to evaluate its effectiveness and to test the data it generates. Moreover, side-lining TNR has done little to settle community concern regarding the management of free-roaming cats. Ultimately, neither culling, nor TNR on their own, are likely to provide an effective solution to the problem of free-roaming cats in Australia. However, management plans will be more successful if they employ a variety of control and eradication methods, as well as engage meaningfully with the ethical, social and cultural dimensions of unwanted species^173^. In the case of TNR, this calls for government facilitating a method that has already demonstrated success at the community level, but which, in an official capacity, has been rebuffed.

## Author Contributions

The author confirms being the sole contributor of this work and has approved it for publication.

### Conflict of Interest Statement

The author declares that the research was conducted in the absence of any commercial or financial relationships that could be construed as a potential conflict of interest.

## Footnotes

^1^RSPCA (1); Prevention of Cruelty to Animals Act 1979 (NSW), ss 5, 6 and 9 (proscription of cruelty); Animal Welfare Act 1985 (SA) s 13 (proscription of ill-treatment); Animal Welfare Act 1992 (ACT) s 6B (duty).

^2^RSPCA (1), 6.

^3^Department of the Environment, *Threat Abatement Plan for Predation by Feral Cats*, Commonwealth of Australia, 2015, 5-7 indicates threats to 74 species of mammals, 40 birds, 21 reptiles, and 4 amphibians. Available online at: http://www.environment.gov.au/system/files/resources/78f3dea5-c278-4273-8923-fa0de27aacfb/files/tap-predation-feral-cats-2015.pdf

^4^Discussion in Part 4.1 of this article.

^5^Low (3); Predation by cats is listed as a threatening process pursuant to the *Environment Protection and Biodiversity Conservation Act 1999* (Cth) s18. The current list of key threatening processes is maintained by Department of the Department of Environment and Energy. Available online at: http://www.environment.gov.au/cgi-bin/sprat/public/publicgetkeythreats.pl; Department of the Environment, *Threat Abatement Plan for Predation by Feral Cats*, above n^3^, 8; Predation by free-roaming cats is also listed as a threatening process pursuant to the *Biodiversity Conservation Act 2016* (NSW) Schedule 4, “Predation by the Feral Cat Felis catus (Linnaeus, 1758);” (4).

^6^Trigger and Mulcock (5); TNR involves capturing free-roaming cats “sterilising them and returning them to the place where they were found”, Paterson (2), 170.

^7^Discussion in part 2.1 of this article.

^8^Discussion in part 3 of this article.

^9^For example, *Game Protection Act* 1866 (NSW), sections 5–7, First and Second Schedules, protected introduced species, such as, pheasants, partridges and deer by having closed hunting seasons. In New South Wales, deer are now listed as key threatening process, pursuant to Schedule 4 of the Biodiversity Conservation act 2016, as are rabbits, goats, foxes, and cats; The Australian Government, has developed a strategy for reducing camel numbers—Department of Sustainability, Environment, Water, Population, and Communities, *National Feral Camel Action Plan: A National Strategy for the Management of Feral Camels in Australia*, (2010) available from http://www.environment.gov.au/system/files/resources/2060c7a8-088f-415d-94c8-5d0d657614e8/files/feral-camel-action-plan.pdf

^10^Historical newspapers were sourced through the “Trove” site of the Australian National Library. The website may be accessed from https://trove.nla.gov.au/

^11^Discussion in part 4.1 of this article.

^12^Department of the Environment, *Threat Abatement Plan for Predation by Feral Cats*, Commonwealth of Australia, 2015, above n^3^, 6.

^13^Ibid, 22.

^14^Abbott has extensively researched the manner of introduction and spread of the cat, including theories that cats were introduced prior to European settlement in 1788; (12) Abbott (6), 4; (13).

^15^Smith (14); Abbott (6), 1; (15).

^16^Abbott (6), 4.

^17^*Dog Nuisance Act*, II GEO IV No 8 (1830)—An Act for abating the Nuisance occasioned by the great number of Dogs which are loose in the Streets of the Towns of Sydney, Parramatta, Liverpool and Windsor in the Colony of New South Wales, s 1; *Native Dogs Destruction Act* 1852 (NSW), sections 1 and 3; *Pastures and Stock Protection Act 1880* (NSW), recital and section 1; *Native Dogs Destruction and Poisoned Baits Act 1901* (NSW), sections 3 and 14; *Pastures Protection Act 1902* (NSW) sections 4 and 58; *Pastures Protection Act 1912* (NSW) section 4; *Birds and Animals Protection Act 1918*, section 3, sections 5-7, First and Second Schedule; Smith (14), 294.

^18^For example, *Pastures and Stock Protection Act 1880* (NSW), ss 8, 14 and 24; *Rabbit Nuisance Act 1883* (NSW) 22 7-12; *Rabbit Act 1902* (WA), s 27-34; *Rabbit Act 1913* (Qld), s 9; *Rabbits Destruction Act 1935* (Tas), ss22-26; *Royal Commission of Inquiry into Schemes for Extermination or Rabbits in Australasia* (Progress Report, Minutes of Proceedings) Government Printer (1890).

^19^Dunlap (9), 82; for example, the *Rabbit Nuisance Act*, An Act to provide for the Abatement of the Rabbit Nuisance 1883 (NSW), provided in sections 4 and 5 that inspectors had power to enter onto crown land to destroy rabbits but did not oblige inspectors or the crown to destroy rabbits.

^20^Stodart and Parer (16).

^21^Dunlap (9), King (18).

^22^*Rabbit Nuisance Act 1883* (NSW), ss 31, 32; *Rabbits Destruction Act 1889* (Tas), s24; *Pastures Protection Act 1902* (NSW), ss 24, 46; *Rabbit Act 1901* (NSW), s46; *Rabbit Act 1902* (WA), s 32; *Pastures Protection Act 1912* (NSW), s 69.

^23^Ibid.

^24^For example, Proclamation by His Excellency, the Right Honorable Lord Augustus William Frederick Spencer Loftus, declared cats as the enemy of the rabbit for a number of Electoral Districts, including, Albury, the Hume, Murrumbidgee, the Murray and Bourke, *New South Wales Government Gazette*, Tuesday July 31, 1883, page 4130; Proclamation by His Excellency, the Right Honorable Henry Robert Brand, Viscount Hampden, that the Iguana was natural enemy of the rabbit within the land district of Boorowa, *New South Wales Government Gazette*, Wednesday, 16 December, 1896, page 9063; Proclamation by His Excellency, Knight Commander of the Bath, Harry H. Rawson, that the iguana, native cat, tiger cat, ferret, mongoose, and stoat were natural enemies of the rabbit within the state of New South Wales, *New South Wales Government Gazette*, Saturday, 29 November, 1902, page 692 (supplement).

^25^“Protection of Cats”, *Albury Banner and Wodonga Express (NSW)*, Friday 18 November 1904 p 29, with respect to the Gundagai region.

^26^“News Notes”, *Macleay Argus (Kempsey, NSW)*, Wednesday 14 September 1892, p 5.

^27^“Rabbits Increasing”, *Bathurst Free Press and Mining Journal*, Tuesday 11 August, 1896, p 2.

^28^For example, 400 cats were transported to Bourke in 1886 to be distributed throughout the region, Denny and Dickman (15), 4–5; 300 cats were released at one location, in Eyre, Stodart and Parer (16), 15.

^29^“News Notes”, above n^26^, 5.

^30^“The Useful Cat”, *Bundarra and Tingha Advocate (NSW)*, Saturday 25 November 1905 p. 2. This statement is attributed to E W Proctor, an Inspector of Stock.

^31^“Agricultural Notes”, *The Leader (Melbourne, Vic)*, Saturday 20 March 1886 p. 9.

^32^“Rabbits and Remedies, the Balance of Nature”, by “Gossip”, *The Sydney Stock and Station Journal (NSW)*, Friday 11 May 1917 p. 3.

^33^“When you are Kept Awake”, *The Armidale Chronicle (NSW)*, Saturday 30 March 1907 p 8.

^34^“Rabbits and Remedies, the Balance of Nature”, by “Gossip”, above n^32^, 3; “When you are Kept Awake”, *The Armidale Chronicle (NSW)*, above n^33^, 8.

^35^Untitled, *The Hay Standard and Advertiser for Balranald, Wentworth, Maude (Hay, NSW)*, Saturday 16 May 1891 p. 2.

^36^Ibid.

^37^“Agricultural Notes”, above n^31^, 9.

^38^“Notes and News”, *Australian Town and Country Journal (Sydney)*, Saturday 16 October 1886, p. 28; “Wild Cat Legislation”, *The Newsletter: an Australian Paper for Australian People (Sydney, NSW)*, Saturday 4 November 1905, p. 15; “Nature is too Slow”, *The Sydney Stock and Station Journal (NSW)*, Friday 13 July 1917, p. 4.

^39^Denny and Dickman (15), 5.

^40^“Notes and News”, above n^38^, 28.

^41^“Wild Cat Legislation”, above n^38^, 15; “Nature is too Slow”, above n^38^, 4.

^42^For example, “A New Cat that eats Lambs”, *The Newsletter: an Australian Paper for Australian People (Sydney)*, Saturday 27 December 1913, p. 5; “Nature is too Slow”, above n^38^, 4.

^43^“A new Cat that eats Lambs”, above n^42^, 5.

^44^For example, ““A new Cat that eats Lambs”, above n^42^, 5; ““Nature is too Slow”, above n^38^, 4.

^45^Discussion in part 2.2 of this article.

^46^“The Cat Problem in Australia”, *Sunday Times (Perth)*, Sunday 22 December 1912 p. 8; “The Cat Problem”, *The Kadina and Wallaroo Times (SA)*, Wednesday 29 January 1913 p. 4.

^47^“The Cat Problem”, above n^46^, 4.

^48^“The Natural Enemy”, *Daily Telegraph (Launceston, Tasmania)*, Saturday 17 March 1894 p. 7.

^49^Abbott WE. *The Rabbit Pest and the Balance of Nature*, cited in Smith (14), 295.

^50^Dunlap (9), 2, 141.

^51^King (18), 54.

^52^Dunlap (9), 87.

^53^“Rabbit Menace” letter to the editor by “one who has suffered”, *The West Australian (Perth)*, Thursday 2 May 1935, p. 10.

^54^Ibid.

^55^Ibid; “Foxy Ways”, *Smith's Weekly (Sydney, NSW)*, Saturday 24 March 1923 p. 23.

^56^“Rabbit Menace”, letter to the editor by “one who has suffered”, above n^53^, 10; “Foxy Ways”, above n^55^, 23.

^57^“Foxy Ways” above n^55^, 23.

^58^*Dog Nuisance Act 1830* No 9a (NSW) (An Act for abating the Nuisance occasioned by the great number of Dogs which are loose in the Streets of the Towns of Sydney Parramatta, Liverpool and Windsor in the Colony of New South Wales); from Tasmania, the *Rabbits Destruction Act 1882* (46 Vic, No. 35); the *Rabbit Boards Act 1896* (Qld); the *Pastures and Stock Protection (Rabbit) Act 1900* (NSW); the *Vermin Act* 1918 (WA); the *Stock Routes Improvement and Animal and Vegetable Pests Destruction Act 1936* (Qld); and the *Pastoral Land Act 2011* (NT); *Local Land Services Act 2013* (NSW), ss 130 and 142 [now repealed and subsumed into the *Biosecurity Act 2015*(NSW)]; *Land Protection (Pest and Stock Route Management) Act 2002* (Qld), sections 36–38.

^59^Ibid.

^60^*Territory Parks and Wildlife Conservation Act 1977* (NT), ss 47–51.

^61^Regulations under the Territory Parks and Wildlife Conservation Ordinance (1978), clause 5, Schedule 3.

^62^*Convention on Biological Diversity 1992*, Article 8(h). The Convention was adopted 5 June 1992, [1993] ATS no 32 (entered into force 29 December 1993). The convention had 196 Parties as of November 2018.

^63^*Environment Protection and Biodiversity Conservation Act 1999* (Cth), ss 183, 188, 528.

^64^*Environment Protection and Biodiversity Conservation Act 1999* (Cth), s183, the list is maintained by Department of the Department of Environment and Energy. Available online at: http://www.environment.gov.au/cgi-bin/sprat/public/publicgetkeythreats.pl

^65^For example, the Blue Mountains regional strategy deals with plant and animal pests, including lantana, foxes, wild dogs, and cats. Office of the Environment and Heritage, Regional Pest Management Strategy 2012–2017: Blue Mountains Region, (2013) 45–46. Available online at: http://www.environment.nsw.gov.au/research-and-publications/publications-search/regional-pest-management-strategy-2012-2017-blue-mountains-region

^66^Department of the Environment, *Threat Abatement Plan for Predation by Feral Cats*, Commonwealth of Australia, 2015, above n^3^. Two prior plans had been made in 1999 and 2008: Environment Australia, Biodiversity Group, *Threat Abatement Plan for Predation by Feral Cats*, (1999); (21).

^67^*Game and Feral Animal Control Act 2002* (NSW), ss 5, 16, Schedule 3.

^68^*Pest Plants and Animals Act 2005* (ACT), ss 16, 19–20.

^69^ACT Government, Environment and Sustainable Development, *ACT Pest Animal Management Strategy, 2012-2022*, (22), 70.

^70^Ibid.

^71^Discussion in part 4.1 of this article.

^72^*Biosecurity Act* 2015 (NSW) s 13, definition of biosecurity risk; *Biosecurity Act 2015* (Cth) contains numerous provisions, where the concept can vary, however Chapter 3 deals with biosecurity risks deriving from imported goods and Chapter 4 manages biosecurity risks with respect to conveyances and vehicles entering Australia.

^73^Denny and Dickman (15), 10.

^74^*Biosecurity Act 2014* (QLD), ss 42–45.

^75^*Biosecurity Act 2014* (QLD), s 43 (1) prohibits the release or distribution of category 3 restricted matter, which in accordance with 43(3) includes giving it to another person or releasing it into the environment; s 44 obliges the holders of category 7 restricted matter to kill or destroy it; s 45 (1) (a) prohibits moving a category 5 restricted matter, while s 45 (1) (c) prohibits feeding a category 6 restricted matter.

^76^*Biosecurity Act 2015* (NSW), ss s 12, 151.

^77^Unattributed author, news story, “Couple to Sue over Savannah Cat Ban,” Sydney Morning Herald, 4 August, 2008. Available online at: https://www.smh.com.au/national/couple-to-sue-over-savannah-cat-ban-20080804-3pi0.html

^78^Mellor and Littin (24); generally, Thiriet D. Flying Fox Conservation Laws, Policies and Practices in Australia - A Case Study in Conserving Unpopular Species, (8), 161.

^79^Dunlap (9), 273.

^80^Paterson (2), 170.

^81^For example, Trigger and Mulcock (5); Paterson (2), 170.

^82^Denny and Dickman (15), 1-2.

^83^Paterson (2), 170.

^84^Department of the Environment. *Threat Abatement Plan for Predation by Feral Cats*. Commonwealth of Australia (2015) above n^3^, 3.

^85^Ibid, 10.

^86^Australian Government (27); Department of the Environment. *Threat Abatement Plan for Predation by Feral Cats*. Commonwealth of Australia (2015). above n^3^, 8–9.

^87^Australian Government. *Threatened Species Strategy*, above n^86^, 11, details 20 mammal species and 20 bird species.

^88^Ibid, 11, 47, 48, 49–50.

^89^Ibid, 48.

^90^Ibid, 11.

^91^Australian Government. *Progress Report to the Minister for the Environment and Energy*, July 2016–December 2017, 19. Available online at: http://www.environment.gov.au/system/files/resources/12d8cf25-0169-46d6-8c72-dfe204ccf44c/files/threatened-species-strategy-progress-report.pdf

^92^Lynn (4); (29).

^93^Environment Australia, Biodiversity Group. *Threat Abatement Plan for Predation by Feral Cats* (1999), above n^66^, 6, 19; Department of the Environment. *Threat Abatement Plan for Predation by Feral Cats*. Commonwealth of Australia (2015), above n^3^, 9; Doherty et al. (13), 92.

^94^Denny and Dickman (15), 2.

^95^Ibid.

^96^Paterson (2), 170.

^97^Department of the Environment. *Threat Abatement Plan for Predation by Feral Cats*. Commonwealth of Australia, 2015, above n^3^, 20.

^98^Ibid, 23.

^99^Denny and Dickman (15), 2.

^100^Ibid.

^101^Department of the Environment. *Threat Abatement Plan for Predation by Feral Cats*. Commonwealth of Australia (2015), above n^3^, 15.

^102^Australian Government, Department of the Environment (26), 5.

^103^Ibid.

^104^“Rabbit Menace,” letter to the editor by “one who has suffered,” above n^53^, 10; “Foxy Ways,” above n^55^, 23; Denny and Dickman (15), 2.

^105^“When you are Kept Awake,” above n^33^, 8; “Rabbits and Remedies, the Balance of Nature,” by “Gossip,” above n^32^, 3; Denny and Dickman (15), 2.

^106^“Agricultural Notes,” above n^31^, 9; Swarbrick and Rand (28), 3–4.

^107^Trigger and Mulcock (5), 1307.

^108^Smith (14), 301.

^109^Trigger and Mulcock (5), 1307; Swarbrick and Rand (28), 2.

^110^Trigger et al. (31); Department of the Environment. *Threat Abatement Plan for Predation by Feral Cats*. Commonwealth of Australia (2015), above n^3^, 15.

^111^Australian Government, Department of the Environment and Energy. *Frequently Asked Questions, Tackling Feral Cats and Their Impacts*, Office of the Threatened Species Commission, 5, 7. Available online at: https://www.environment.gov.au/system/files/resources/bb591b82-1699-4660-8e75-6f5612b21d5f/files/factsheet-tackling-feral-cats-and-their-impacts-faqs.pdf; also Australian Government, Department of the Environment (26), 6; Department of the Environment. *Threat Abatement Plan for Predation by Feral Cats*. Commonwealth of Australia (2015), above n^3^, 23.

^112^Riley (8), 280.

^113^Lynn (4).

^114^Doherty et al. (13), 84.

^115^Dickman (33); Institute of Wildlife Research University of Sydney (1996). Available online at: https://www.pestsmart.org.au/wp-content/uploads/2010/03/impacts-feral-cats.pdf; Doherty et al. (13), 87-88.

^116^Doherty et al. (13), 94.

^117^Lynn (4).

^118^Crowley et al. (32), 129.

^119^Low T. *Feral Future*, above n^5^, 190–194.

^120^Ibid, 191.

^121^Ibid, 190–194.

^122^Dickman C. Overview of the impacts of feral cats on Australian Native Fauna, (33), parags 3.4, 4.2.

^123^Paltridge (38); Paterson (2), 172.

^124^Mellor and Littin (24), 44.

^125^Department of the Environment. *Threat Abatement Plan for Predation by Feral Cats*, Commonwealth of Australia, 2015, above n^3^, objective 1, action 1.3 and 1.4, p 15-16, objective 2, action 2.3, p 19, objective 3, action 3.4, p. 22.

^126^Australian Government. *Threatened Species Strategy*, above n^86^, 41, 63.

^127^Environment Australia, Biodiversity Group. *Threat Abatement Plan for Predation by Feral Cats* (1999), above n^66^, 17.

^128^Ibid.

^129^Department of the Environment, Water, Heritage and the Arts. *The Threat Abatement Plan for Predation by Feral Cats* (2008), (21), objective 4, p 8.

^130^Department of the Environment. *Threat Abatement Plan for Predation by Feral Cats*. Commonwealth of Australia (2015), above n^3^, 20.

^131^Generally, Riley (8).

^132^Department of the Environment. *Threat Abatement Plan for Predation by Feral Cats*. Commonwealth of Australia (2015), above n^3^, 15-17.

^133^Australian Government, Department of the Environment (26), 16.

^134^Doherty et al. (13), 90.

^135^Ibid.

^136^“The Cat Problem”, above n^46^, 4.

^137^Ibid.

^138^Generally, Spehar and Wolf (40); Swarbrick and Rand (28), 2; Andersen et al. (41).

^139^Generally, Spehar and Wolf (40), 1-2.

^140^Australian Pet Welfare Foundation, runs a Community Cat Program https://www.petwelfare.org.au/community-cat-programs/

^141^Campus Cat Coalition, information available from http://www.campuscats.org.au/

^142^Department of the Environment. *Threat Abatement Plan for Predation by Feral Cats*. Commonwealth of Australia, 2015, above n^3^, 10.

^143^Ibid, 23.

^144^Ibid.

^145^*Prevention of Cruelty to Animals Act 1979* (NSW), s 11.

^146^*Prevention of Cruelty to Animals Act 1979* (NSW), s 4.

^147^*Domestic Animals Act 1994* (Vic), s 33; *Prevention of Cruelty to Animals Act 1986* (Vic), s 9 (1)(h).

^148^Discussion in part 3 of this paper.

^149^Animal Ethics committees are established pursuant to the National Health and Medical Research Council. (45); Examples of specific implementation at the state level include Animal Research Act 1985 (NSW), ss 13-16 and the Prevention of Cruelty to Animals Act 1986 (Vic) s 86.

^150^By way of illustration, *Research and Integrity Policy at the University of Technology Sydney*, section 3.2, available from http://www.gsu.uts.edu.au/policies/research-ethics-integrity-policy.html#principles; *Macquarie University Code for the Responsible Conduct of Research*, section 17.1, available from https://staff.mq.edu.au/work/strategy-planning-and-governance/university-policies-and-procedures/policies/responsible-conduct-of-research/media/The-Macquarie-University-Code-for-the-Responsible-Conduct-of-Research_June2017.pdf

^151^Animal Welfare (Population Control Programs) Bill 2014, text of bill available from https://www.parliament.nsw.gov.au/bill/files/2821/First%20Print.pdf; explanatory notes available from https://www.parliament.nsw.gov.au/bill/files/2821/XN%20Animal%20Welfare.pdf; second reading speech available from, https://www.parliament.nsw.gov.au/bill/files/2821/2R%20Animal%20Welfare.pdf

^152^Ibid.

^153^Gotsis (25), 10, 14.

^154^Ibid.

^155^Ibid, 14.

^156^Swarbrick and Rand (28), 3-4.

^157^Ibid.

^158^Andersen et al. (41), 1875; Paterson (2), 170.

^159^Paterson (2), 172.

^160^Tan et al. (42), 15.

^161^Sherley (48); 1080 is the main poison used to kill foxes, for the government's position see NSW Government, Local Land Services, Foxes Factsheet, http://www.lls.nsw.gov.au/biosecurity/pest-control/foxes

^162^Spehar and Wolf (40), 1–2.

^163^Ibid.

^164^Ibid.

^165^Tan et al. (42), 19.

^166^Schaffner JE. *Community Cats: Changing the Legal Paradigm for The Management of So-Called “Pests”*. 67 *Syracuse Law Review* (2017) 71, 90.

^167^Andersen et al. (41), 1871, 15.

^168^Crowley et al. (32), 122.

^169^Ibid.

^170^Ibid, 136.

^171^Nagy K, Johnson PD II. Introduction. In: Nagy K, Johnson PD II, Editors. *Trash Animals: How We Live With Nature's Filthy, Feral, Invasive, and Unwanted Species*. University of Minnesota Press (2013). p. 6–9.

^172^Generally, Paterson (2); Doherty et al. (13), 93; Department of the Environment. *Threat Abatement Plan for Predation by Feral Cats*. Commonwealth of Australia (2015), above n^3^, 23.

^173^Denny and Dickman (15), 1–2; Doherty et al. (13), 93.
